# Supplementary material for: The Sorting Nexin Genes ChSNX4 and ChSNX41 Are Required for Reproductive Development, Stress Adaption and Virulence in Cochliobolus heterostrophus
Source: J Fungi (Basel). 2022 Aug 15;8(8):855. doi: 10.3390/jof8080855 (PMC9410474; doi:10.3390/jof8080855)
Supplement: Supplementary file 1 [file jof-08-00855-s001.zip › jof-1847585-supplementary.pdf]

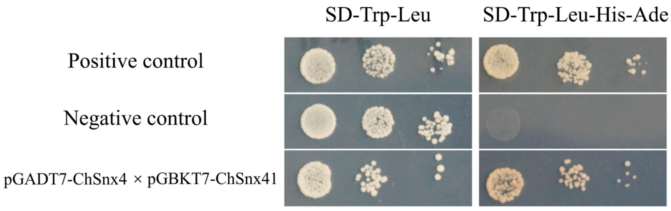

Figure S1. Yeast two-hybrid analyses for interaction between ChSnx4 and ChSnx41. Yeast cells transformed with bait and prey constructs indicated were examined on SD-Trp-Leu-His-Ade plates. The interaction between pGBKT7-53 and pGADT7-T and between pGBKT7-Lam and pGADT7-T were used as positive control and negative control, respectively.

Table S1. Primers used in this study.

**Primer**	**Sequence 5' to 3'**	**Description**	**Size of PCR product (kb)**	**Purpose**
Chsnx4-FP1	CCTGTCGCAGGCATCAAG	5' flanking region of *SNX4,* forward primer	0.82	Delete *SNX4*
Chsnx4-RP1	TCCTGTGTGAAATTGTTATCCGCT AACTCGCCCAAGCAAACG	5' flanking region of *SNX4,* reverse primer with hygB gene tail		
Chsnx4-FP2	GTCGTGACTGGGAAAACCCTGGC GTTAGGCGTAATGAGTGGC	3' flanking region of *SNX4,* forward primer with hygB gene tail	0.81	
Chsnx4-RP2	AGAAGGGTTGGTTGTTGG	3'flanking region of *SNX4,* reverse primer		
M13R	AGCGGATAACAATTTCACACAGG A	Forward primer to amplify hygB	2.5	
M13F	CGCCAGGGTTTTCCCAGTCACGA C	Reverse primer to amplify hygB		
NLC37	GGATGCCTCCGCTCGAAGTA	Located at hygB, reverse primer	2.1	*SNX4* deletion verification
Chsnx4-UF	GCGACCTGCTGAGGAAGAAT	Upstream verification forward primer of *SNX4*, pair with NLC37		
NLC38	CGTTGCAAGACCTGCCTGAA	Located at hygB, forward primer	2.7	
Chsnx4-DR	CATCTCCTTTAGTCTTGGCA	Downstream verification reverse primer of *SNX4*, pair with NLC38		
Chsnx4-F	GTCGGCAGCCCACAGAAA	Forward primer to	1.0	

		amplify *SNX4*		
Chsnx4-R	GCCCGTCAGACGCTCAAT	Reverse primer to amplify *SNX4*		
Chsnx4-FP1	CCTGTCGCAGGCATCAAG	*SNX4* including 5' flanking and 3' flanking, forward primer	3.5	*SNX4* complementation
Chsnx4-C1	CACTGGAACAACTGGCATGAGAAGGGTTGGTTGTTGG	*SNX4* including 5' flanking and 3' flanking, reverse primer		
DW69	CATGCCAGTTGTTCCAGTG	Forward primer to amplify *npt*II	2.7	
DW70	ACCTCTAAACAAGTGTACCTG	Reverse primer to amplify *npt*II		
Chsnx4-C2	CAGGTACACTTGTTTAGAGGTATACCCGTTAGATGACAAGAT	Further downstream of 3' flanking of *SNX4*, forward primer	0.6	
Chsnx4-C3	TGTCGACGCTCGAGTATTTG	Further downstream of 3' flanking of *SNX4*, reverse primer		
Chatg20-FP1	TTGGCATCACCTACAAGCAG	5' flanking region of *SNX41,* forward primer	0.65	Delete *SNX41*
Chatg20-RP1	TCCTGTGTGAAATTGTTATCCGCTTGGGTGTTGAGGATCTAGGG	5' flanking region of *SNX41,* reverse primer with hygB gene tail		
Chatg20-FP2	GTCGTGACTGGGAAAACCCTGGCGGGTCTTTGTCGCCAACATCT	3' flanking region of *SNX41,* forward primer with hygB gene tail	0.7	
Chatg20-RP2	TCTCACTCGTCGCATGATTC	3'flanking region of *SNX41,* reverse primer		
M13R	AGCGGATAACAATTTCACACAGGA	Forward primer to amplify hygB	2.5	
M13F	CGCCAGGGTTTTCCCAGTCACGAC	Reverse primer to amplify hygB		
NLC37	GGATGCCTCCGCTCGAAGTA	Located at hygB, reverse primer	2.1	*SNX41* deletion verification
Chatg20-UF	ACCGTGTTGCGTAGAGTGGA	Upstream verification forward primer of *SNX41*, pair with NLC37		
NLC38	CGTTGCAAGACCTGCCTGAA	Located at hygB, forward primer	2.7	
Chatg20-DR	AAACTCGCAAGGTCCGCACG	Downstream verification reverse		

		primer of *SNX41*, pair with NLC38		
Chatg20-F	ATGTGGGACGACGAGGAC	Forward primer to amplify *SNX41*	1.75	
Chatg20-R	CGTGATGGAACCGAATAG	Reverse primer to amplify *SNX41*		
Chatg20-FP1	TTGGCATCACCTACAAGCAG	*SNX41* including 5’ flanking and 3’ flanking, forward primer	3.9	*SNX41* complementation
Chatg20-C1	CACTGGAACAACTGGCATGTCTC ACTCGTCGCATGATTC	*SNX41* including 5’ flanking and 3’ flanking, reverse primer		
DW69	CATGCCAGTTGTTCCAGTG	Forward primer to amplify *npt*II	2.7	
DW70	ACCTCTAAACAAGTGTACCTG	Reverse primer to amplify *npt*II		
Chatg20-C2	CAGGTACACTTGTTTAGAGGTCTT GTATGGCCGTTTGCG	Further downstream of 3’ flanking of *SNX41*, forward primer	0.73	
Chatg20-C3	TGGCGTTATGGTTAGTTTCT	Further downstream of 3’ flanking of *SNX41*, reverse primer		
